# Secondary involvement of gallbladder by acute lymphoblastic leukemia presenting clinically as cholecystitis in a young patient: a case report

**DOI:** 10.1186/s12957-023-02956-4

**Published:** 2023-02-27

**Authors:** Shabina Rahim, Zubair Ahmad, Qurratulain Chundriger, Arsalan Ahmed, Natasha Ali, Jamshid Abdul-Ghafar

**Affiliations:** 1 Department of Pathology and Laboratory Medicine, Aga Khan University Hospital, Karachi, Pakistan; 2 Department of Pathology and Clinical Laboratory, French Medical Institute for Mothers and Children (FMIC), Kabul, Afghanistan

**Keywords:** B-Lymphoblastic leukemia/lymphoma, Gallbladder, Leukemia

## Abstract

**Background:**

Primary lymphoma of the liver, gallbladder, and extrahepatic bile ducts or secondary involvement of these organs by leukemia is exceedingly rare. Patients with primary lymphoma or leukemic involvement of the biliary tract and liver often present with symptoms and signs of biliary tract obstruction or inflammation.

**Case presentation:**

We present a case of a 24-year-old male with biliary tract symptoms who underwent laparoscopic cholecystectomy. His precholecystectomy complete blood count performed on the same morning showed 72% lymphocytes while peripheral blood smears showed approximately 15% blasts. Surgeon went ahead with the procedure. Imaging done prior to surgery showed thickened gallbladder, while the liver, biliary tract, and pancreas did not show any thickening or mass lesion. However, the liver was enlarged. Grossly, the gallbladder wall did not show any stones or discrete mass involving the wall. Instead, there was subtle thickening of the gallbladder wall due to diffuse infiltration by the leukemic infiltrate. This lymphoid population reacted with PAX-5 and TdT immunohistochemical antibodies in a diffuse manner confirming precursor B-cell origin. This patient was found to have B-lymphoblastic leukemia involving his bone marrow on further clinical and diagnostic workup. Patient responded well to chemotherapy and is currently on maintenance treatment. He is well 1.5 years after his diagnosis.

**Conclusion:**

This case highlights a unique and rare scenario where a previously undiagnosed and unsuspected hematologic malignancy initially presented with clinical features of a chronic inflammatory condition involving an abdominal organ owing to secondary involvement by the malignant infiltrate.

## Introduction

Primary lymphoma of the liver, gallbladder, and extrahepatic bile ducts or secondary involvement of these organs by leukemia is exceedingly rare. Both lymphoid and myeloid leukemia have been reported to involve the gallbladder. Most patients with primary or secondary involvement of the gallbladder present with symptoms mimicking cholecystitis. Patients with lymphoma/leukemic involvement of biliary tract and liver often present with symptoms and signs of biliary tract obstruction such as cholestatic jaundice and liver failure. Clinical presentation, radiological findings, and pre-operative findings are not often helpful in distinguishing primary or secondary involvement of these organs by hematolymphoid malignancies and preoperative diagnosis is extremely difficult [1–5]. A gallbladder with primary lymphoma or secondarily involved by a leukemic infiltrate often shows diffuse thickening of the wall mimicking cholecystitis [6–20].

Herein, we report a case of acute lymphoblastic leukemia (ALL) in a young male involving the gallbladder secondarily. We describe the clinical, radiological, gross, microscopic, and immunohistochemical (IHC) findings and present a detailed review of the published literature.

## Case presentation

### Clinical presentation

A 24-year-old male presented with severe right upper quadrant abdominal pain for 3 months. There was no significant past medical history. He was vitally stable and general examination was unremarkable except for epigastric tenderness. His laboratory findings revealed abnormal liver function tests. Imaging studies were suggestive of cholelithiasis with an evolving cholecystitis. The liver was enlarged. An elective laparoscopic cholecystectomy was planned. His initial peripheral blood smears done on the day of the surgery showed 72% lymphocytes on differential leukocyte count, although total white cell count was 3.9 × 10^9^E/L. Peripheral smear showed approximately 15% blasts. Hemoglobin was 9.4 gm/dL, while platelet count was low 100 × 10^9^E/L. The surgeon decided to proceed with the procedure as he had no suspicion that the gallbladder was infiltrated by a neoplastic lesion and believed that the blast cells seen on complete blood count were an independent finding from cholelithiasis and cholecystitis.

### Radiological findings

Pre-cholecystectomy ultrasound of liver and gallbladder showed moderately distended and thickened gallbladder measuring 80 × 35 mm and containing tiny stones mixed with sludge. Wall thickness was 7 mm. The liver was enlarged and showed mild increase in parenchymal echogenicity suggesting mild fatty infiltration. No focal lesion was seen. No intra- or extrahepatic biliary dilation was present. The common bile duct (CBD) and portal vein were unremarkable. Magnetic resonance cholangiopancreatography (MRCP) showed slightly edematous, distended, and thick-walled gallbladder with sludge and stones. Ultrasound, MRI, and MRCP findings were suggestive of cholelithiasis with evolving cholecystitis. There was no evidence of any intra or extra hepatic biliary dilatation. The right and left hepatic ducts and cystic duct appeared unremarkable. Post-cholecystectomy ultrasound, MRI, and MRCP showed no focal lesion or distortion in liver, biliary tract, or spleen. However, the liver appeared enlarged. No evidence of bilateral pleural effusion or ascites was noted. He underwent laparoscopic cholecystectomy.

### Operative findings

On laparoscopic cholecystectomy, the gallbladder was distended and was adherent to the liver. The cystic duct was short and wide. Dissection was difficult due to adhesions and hepatomegaly. The liver appeared inflamed and friable; peri-hepatic fluid was present. Adhesiolysis was performed and dissection was continued over the liver bed. The gallbladder was dissected from the gallbladder fossa. Some bleeding was encountered due to traction on the liver.

### Pathological analysis

**Fig. 1:**
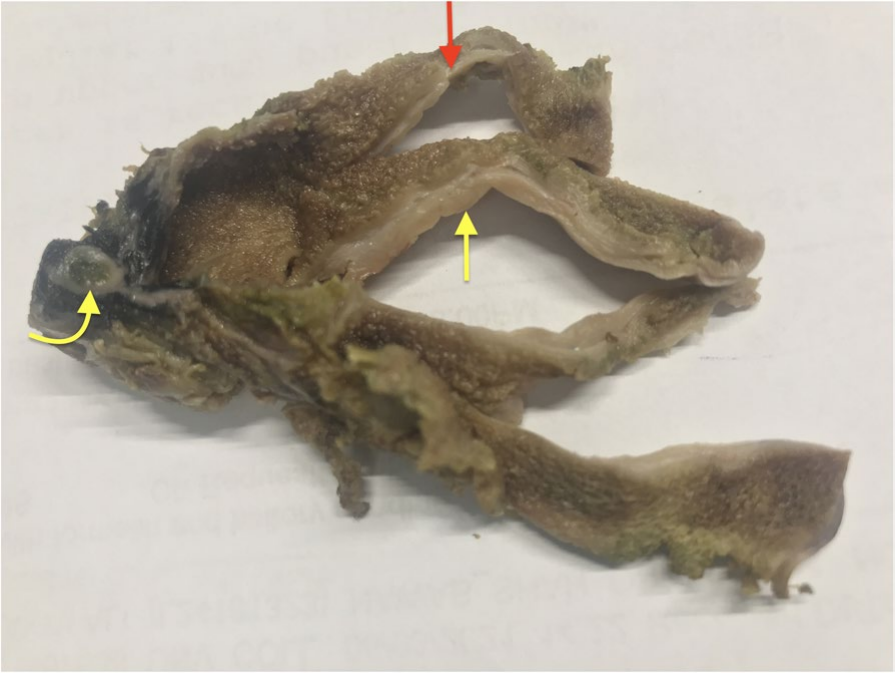
Fundal wall showed an average thickness of 5 mm, red arrow shows thickening of the wall as compared to uninvolved area marked by yellow arrow. Curved yellow arrow cystic duct margin

Grossly, the gallbladder measured 7 cm in length and 2 cm in diameter. The serosal surface was smooth. The fundal wall showed an average thickness of 5 mm, with very subtle thickening at some places (Fig. 1, red arrow shows thickening of the wall as compared to uninvolved area marked by yellow arrow. Curved arrow points at cystic duct margin). No stones were present in the lumen.

**Fig. 2:**
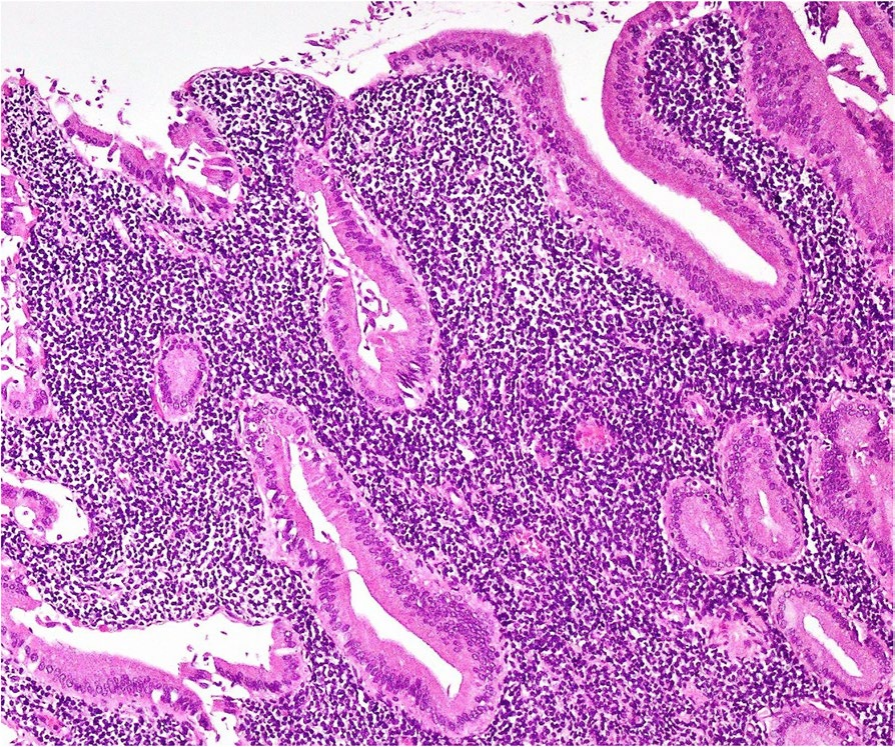
(H&E ×200): Densely cellular atypical lymphoid cell infiltrate composed of small to medium sized cells infiltrating diffusely into the lamina propria, with entrapped crypts, lined by uniform looking columnar biliary epithelial cells

**Fig. 3:**
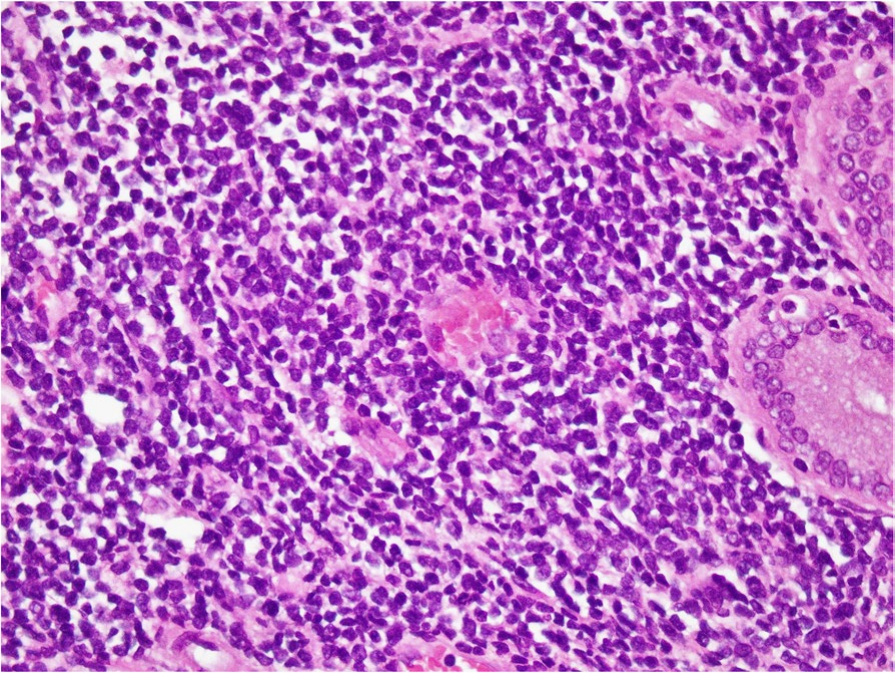
(H&E ×400) High power view showing details of cells, having round to oval, convoluted nuclei, condensed nuclear chromatin, indistinct nucleoli, and scant cytoplasm

**Fig. 4:**
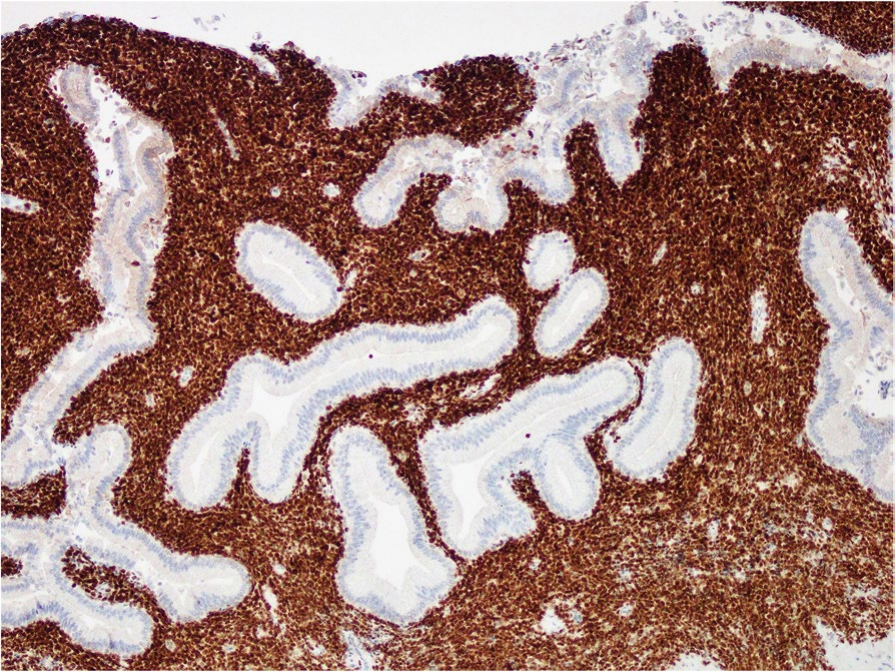
The neoplastic lymphoid cells demonstrated strong diffuse nuclear positivity for PAX-5 immunohistochemical stain

**Fig. 5:**
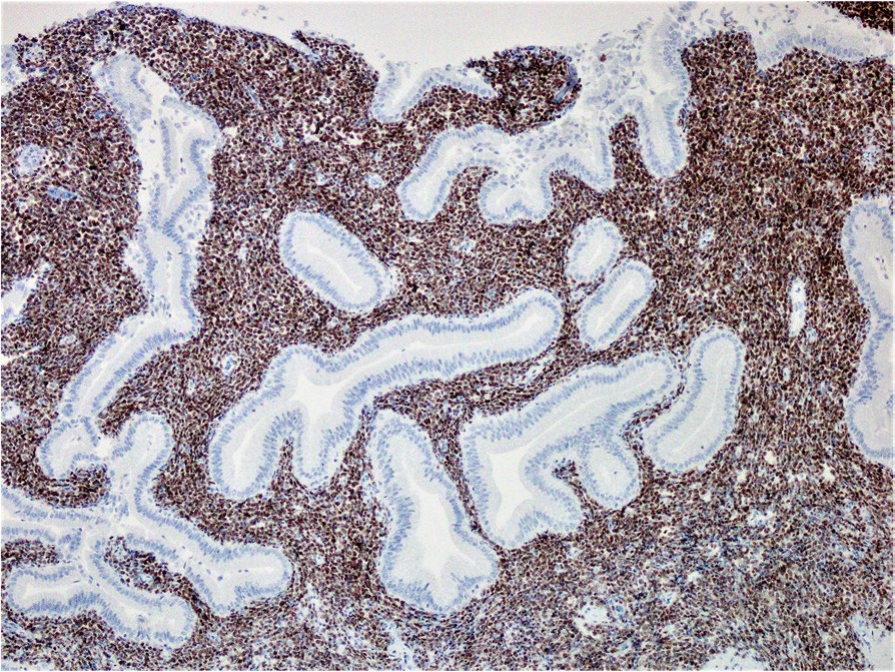
The neoplastic lymphoid cells demonstrated strong diffuse nuclear positivity for Tdt immunohistochemical stain

Microscopic examination of the resected specimen revealed diffuse, densely cellular atypical lymphoid cell infiltrate involving the full thickness of the gallbladder wall. Cystic duct resection margin was involved. The infiltrate was composed of small- to medium-sized cells with round to oval, convoluted nuclei, condensed nuclear chromatin, indistinct nucleoli, and scant cytoplasm (Fig. 2, medium power view of H&E-stained section). Nuclear to cytoplasmic ratio was high (Fig. 3, high power view of H&E-stained section showing cellular details). Numerous mitotic figures were seen. IHC studies were performed, and the neoplastic lymphoid cells demonstrated strong diffuse positivity for PAX-5 (Fig. 4) and terminal deoxynucleotidyl transferase (TdT) (Fig. 5). Ki-67 (Mib-1) proliferative index was raised up to approximately 60% (not shown). The neoplastic cells were negative for Keratin cocktail AE1/AE3, CD20, CD3, CD5, myeloperoxidase (MPO), CD34, and IgG4. Liver biopsy was not performed.

### Final diagnosis and further workup

Based on these findings, a diagnosis of B-lymphoblastic lymphoma/ leukemia (B-LL) was rendered. Peripheral blood film was repeated and bone marrow biopsy was performed post-resection. Peripheral blood film showed normochromic, microcytic red blood cells. Hemoglobin was 7.8 gm/dl. Approximately 80% blast cells were seen. Platelets were low on film. Bone marrow aspirate revealed diffuse infiltration with blast cells which constituted approximately 85% of the total nucleated non-erythroid cell population. Similarly, section of bone trephine also showed hypercellularity (approximately 80 to 85%) and diffuse infiltration by blast cells. Immunophenotyping by flow cytometry performed on bone marrow showed reactivity of blast cell population to pan B-cell markers i.e., CD10, CD19, CD22, and CD79a along with HLA-DR and CD45. This population also showed positivity to CD9, CD58, CD34, and TdT. Overall findings were consistent with B-LL.

### Molecular analysis

ABL1 by FISH/PCR was subsequently performed and was negative. Conventional cytogenetics showed 46 XY chromosomes.

### Clinical course

Our patient responded well to treatment. He received treatment with MRC UK ALL XII protocol. His minimal residual disease after induction phase I was less than 0.01%. He subsequently successfully completed all blocks of treatment till consolidation. During induction phase I, he developed steroid-induced hyperglycemia which reverted with insulin and oral hypoglycemics. Since then, he has not developed any other chemotherapy-related complications. He is currently on maintenance treatment with 6-mercaptopurine and methotrexate with prophylactic trimethoprim/sulphamethoxazole. His last complete blood count (CBC) showed hemoglobin 13.2 gm/dl, white blood cells (WBC) 5.1 × 10^9^E/L, platelets 450 × 10^9^E/L. He is well 2 years after the diagnosis.

## Discussion

Secondary involvement of gallbladder by malignancies is extremely rare and malignant melanomas constitute the most common tumor type [21]. In more than 90% cases, diagnosis is late, often at the terminal stage of malignancy with involvement of multiple organs [22]. Although most cases present as diffuse infiltration, involvement by lymphoma/leukemia may rarely present in the form of true polyps [23].

Over the years, several case reports have documented primary or secondary involvement of liver, gallbladder, and biliary tract by acute and chronic lymphoid [5–15] or acute and chronic myeloid [16–19] leukemia. These are listed in Table 1.

**Table 1:** Primary or secondary involvement of gallbladder, biliary tract, and liver by leukemia/lymphoma

Sr#	Study	Year of publication	Organ involved	Number of cases	Diagnosis
1	Finley et al. [6]	1993	Gallbladder	1	ALL
2	Fidias et al. [1]	1995	Biliary tract	7	All mature B-cell lymphomas
3	Costa et al. [10]	1998	Liver	1	CLL
4	Chim et al. [11]	2001	Gallbladder	1	CLL
5	Bloom et al. [16]	2002	Gallbladder	1	AML
6	Shimizu et al. [17]	2006	Gallbladder	1	AML
7	Dellon et al. [2]	2006	Liver	1	Natural killer-like T cell leukemia/lymphoma
8	Bartley et al. [18]	2007	Gallbladder	1	AML
9	Patel et al. [7]	2009	Gallbladder	1	T-LL
10	Dasanu et al. [12]	2010	Gallbladder	1	CLL
11	Hwang et al. [4]	2010		5	DLBCL (1 case), granulocytic sarcoma (1 case), plasmacytoma (1 case), extranodal marginal zone lymphoma (2 cases)
12	Mani et al. [3]	2011		19	Primary (14): DLBCL (3 cases), FL (3 cases), extranodal marginal zone lymphoma (2 cases), MCL (1 case), B-LL (2 cases), HIV-associated lymphoma (2 cases), T-cell lymphoma (1 case). Secondary (5): B-cell lymphoma (4 cases), CHL (1 case).
13	Rao et al. [13]	2011	Gallbladder	1	CLL
14	Esfahani et al. [14]	2011	Liver	1	CLL
15	Ozawa et al. [8]	2012	Gallbladder	1	B-LL
16	Psarras et al. [5]	2014	Gallbladder	1	B-LL (primary)
17	Azin et al. [19]	2014	Gallbladder	1	AML
18	Mitropoulos et al. [20]	2015	Gallbladder	1	T-LL (primary)
19	Sayyed et al. [9]	2018	Liver	1	ALL
20	Jafroodifar et al. [15]	2021	Gallbladder	1	CLL
21	Present case report, Rahim et al.	2022	Gallbladder	1	B-LL

*ALL* acute lymphoblastic leukemia/lymphoma, *B-LL* B-lymphoblastic leukemia/lymphoma, *T-LL* T-lymphoblastic leukemia/lymphoma, *CLL* chronic lymphocytic leukemia, *AML* acute myeloid leukemia, *FL* follicular lymphoma, *DLBCL* diffuse large B-cell lymphoma, *CHL* classic Hodgkin lymphoma, *MCL* Mantle cell lymphoma

Most cases of leukemic infiltration of gallbladder present with clinicopathological features closely resembling acute and/or chronic cholecystitis [6, 11–13, 16, 17]. Azin et al reported a patient with known AML who received chemotherapy and achieved a morphological free state. He developed signs and symptoms of cholecystitis 2 years later for which he underwent cholecystectomy. Histological examination revealed extensive infiltration by AML [19]. Liver involvement by CLL leading to liver failure has also been reported [10, 14, 15].

Constitutional symptoms in such patients include nausea, vomiting, right upper quadrant and epigastric abdominal pain and cramps, bloating, abdominal swelling, and mass due to enlargement of the liver, spleen and regional lymph nodes, and/or fluid accumulation in the abdominal cavity, persistent weakness and fatigue, weight loss, obstructive jaundice, and gall stones [1–7, 9, 12–14, 16, 17, 19, 20, 22].

In cases where cholecystectomy was performed with suspicion of cholelithiasis and acute or chronic cholecystitis reveals infiltration by lymphoma or leukemia, blood tests (leukocytosis, abnormal liver function tests, raised CA 19-9), bone marrow aspirate, fine needle aspiration cytology (FNAC) of abdominal fluid for malignant cells, and endoscopic ultrasound-guided fine needle aspiration (EUS-FNA) for evaluation of lymph nodes in porta hepatic region should be carried out. In addition, abdominal ultrasound, magnetic resonance imaging (MRI), and computed tomography (CT) scan should be performed to investigate systemic involvement [24].

Leukemic infiltration only rarely produces a discrete focal mass. Even the gross appearance at cholecystectomy is often strongly suggestive of acute and/or chronic cholecystitis [11, 12, 16, 17]. In a series of 19 cases of lymphoma and leukemia involving the gallbladder and extrahepatic bile ducts, 14 were primary and five were secondary. In case of the latter, there was no prior diagnosis of lymphoma and it was diagnosed post-cholecystectomy. These five patients were found to have widespread disease on additional workup. However, these patients came to clinical attention due to involvement of the gallbladder [3]. In most cases of leukemic infiltration, histopathology shows diffuse infiltration of the gallbladder wall by the atypical lymphoid or myeloid infiltrate. IHC is required to rule out poorly differentiated carcinoma [8]. If there is known history of lymphoid or myeloid leukemia, appropriate IHC will resolve the issue. However, when there is no known history of leukemia and gallbladder involvement is the first manifestation of the disease, an extensive IHC panel may need to be performed. However, a CBC may help enormously in such cases in alerting the clinician if the lymphoid or myeloid cell counts are abnormal. A subsequent bone marrow examination supplemented by IHC will confirm the diagnosis. CD15 positivity indicates a myeloid lineage [17]. CD4, CD43, CD45, CD68, MPO, and lysozyme positivity also indicate a myelomonocytic derivation [18]. Positivity for IHC stains CD79a, CD10, CD 4, and TdT is also seen in B lymphoblasts in cases of B-ALL. However, cyclin D1 is negative [9, 15]. T-cell lineage is indicated by positivity for CD1a, CD3, CD4, CD5, CD68, and CD43 [7]. B-cell lineage is indicated by positivity for CD20, BCL2, CD5, CD43, and CD23. Positivity for CD38 may be associated with poor prognosis in cases of CLL if expressed by more than 30% of the atypical B lymphoid cells [14, 20].

Prognosis of B-LL has improved with new chemotherapy. There is a >95% complete remission rate in children compared to 60–85% in adults. Approximately 80% children are cured while cure rate in adults is <50% [25]. T-LL is associated with a higher risk for induction failure and early relapse compared to B-LL [26]. Chronic lymphocytic leukemia (CLL) has a better prognosis than LL [27–29]. Prognosis of AML is poor despite chemotherapy with survival times of only a few months [30, 31]. NK lymphoblastic leukemia /lymphoma is considered indistinguishable from AML both in terms of treatment and prognosis. Prognosis of CML is excellent with tyrosine kinase inhibitor (TKI) therapy. Mortality rates have been reduced to only 2 to 3% per year while 5-year survival rates have increased to 80–95% [32, 33].

The usual chemotherapy regimen for patients with LL includes vincristine, dexamethasone or prednisolone, and an anthracycline drug such as doxorubicin (Adriamycin) or daunorubicin. Some regimens may also include cyclophosphamide, L-asparaginase, and/or high doses of methotrexate or cytarabine (Ara-C) as part of the induction phase. In LL patients whose leukemia cells have the Philadelphia chromosome, a targeted drug such as imatinib (Gleevec) or dasatinib (Sprycel) is often included. For AML, combination of cytarabine with anthracycline (Daunorubicin) has been used for decades. However, several novel targeted therapies are now becoming available. These include hypomethylating agents, drugs inhibiting Hedgehog pathway and drugs modulating TP53 pathway, etc. In addition, allogenic hematopoietic stem cell transplant can be considered for post-remission therapy in patients with adverse risk of relapse. In CML, targeted therapy with imatinib, dasatinib, and nilotinib (Tasigna) is given. In a group of patients with CLL (young, fit with mutated IGHV, without TP53 mutations or deletions in chromosomes 11 or 17), a defined course of therapy with fludarabine, cyclophosphamide, and rituximab has been shown to be of great benefit and many patients achieve durable remissions [28, 34–43].

## Conclusion

Involvement of the gallbladder, biliary tract, or liver by leukemic infiltrate is very rare and documented cases mostly present with symptoms strongly mimicking acute and/or chronic inflammation of the involved organs. Gallbladder involvement usually presents with features suggestive of acute or chronic cholecystitis and diffuse thickening of the wall rather than a discrete mass. When there is no previous diagnosis of leukemia, it is not possible in most cases to make a pre-operative diagnosis and leukemia is only diagnosed on histopathological examination of the resected gallbladder.

## Data Availability

All data generated are included in this article.

## Abbreviations

ALL: Acute lymphoblastic leukemia
AML: Acute myeloid leukemia
CBC: Complete blood count
CBD: Common bile duct
CLL: Chronic lymphoid leukemia
CT: Computed tomography
IHC: Immunohistochemical
MPO: Myeloperoxidase
MRCP: Magnetic resonance cholangiopancreatography
TdT: Terminal deoxynuleotidyl transferase
WBC: White blood cells
